# Prognostic Factors and Incidence for Postictal Agitation After Electroconvulsive Therapy: A Systematic Review and Meta-analysis

**DOI:** 10.1097/YCT.0000000000001032

**Published:** 2024-08-22

**Authors:** Melissa Ertman, Emy S. van der Valk Bouman, Pascal R.D. Clephas, Tom K. Birkenhager, Markus Klimek

**Affiliations:** From the Departments of ∗ Neuroscience; † Anesthesiology; ‡ Psychiatry, Erasmus Medical Center, Rotterdam, the Netherlands.

**Keywords:** electroconvulsive therapy, incidence, meta-analysis, postictal agitation, prognostic factors

## Abstract

Postictal agitation (PIA) is an adverse effect of electroconvulsive therapy (ECT) and is known to predict other side effects of ECT, but inconsistencies in the literature remain regarding PIA prognostic factors and incidence. Therefore, a systematic review and meta-analysis were conducted (1) to identify prognostic factors for PIA following ECT and (2) to elucidate the diverse incidences of PIA following ECT based on demographic and clinical characteristics. Specifically, electronic databases were searched for retrospective observational studies and randomized controlled trials (RCTs) that objectively reported PIA incidence. Additional inclusion criteria encompassed studies involving patients 18 years or older and allowed for the extraction of PIA prognostic factors. This resulted in the inclusion of 21 articles with 66,047 patients in total. A total of 35 prognostic factors were identified for PIA after ECT, consisting of 8 anesthesia-related, 19 patient-related, and 8 ECT-related prognostic factors. A meta-analysis was conducted for 7 prognostic factors. None of the prognostic factors demonstrated a significant effect on reducing or increasing PIA incidence. Mean PIA was 13.9% (18.0% adjusted) at the patient level and 12.4% (16.5% adjusted) at the session level. Overall risk of bias was generally moderate to low, except in the outcome measurement domain, where 43% of the studies had a high risk of bias. Although none of the prognostic factors in meta-analysis were significant, several other prognostic factors consistently indicated increased or decreased risk, providing direction for future research. A scarcity of (high-quality) data emphasizes the need for additional research on this topic to be conducted.

Postictal agitation (PIA) is a phenomenon that occurs after an electroconvulsive therapy (ECT)–induced seizure and is often characterized by anxiety, disorientation, clouded consciousness, motor agitation, nonresponse to verbal commands, and sometimes violent behavior and can last up to an hour after ECT treatment.^1,2^ PIA is not only a dangerous adverse effect of ECT as patients can harm themselves in the postanesthesia care unit,^3^ but its presence and duration can also be a predictor of other common ECT-related side effects, including amnesia.^4^

Incidence rates for PIA vary from about 8% to 65% of patients who undergo ECT treatment, depending on how PIA is defined, the exact procedure used for ECT, and the patient population involved.^2,5–7^ Although the term “postictal agitation” is commonly used, clinicians and researchers also refer to the post-ECT phenomenon as delirium, confusion, restlessness, and disorientation and diagnose it based on differing criteria and degrees of severity. These varying definitions and wide range of incidence create a challenge in understanding incidence rates, implications, risk factors, and mechanisms for PIA, which are crucial factors for its prevention and treatment.

Prognostic factors are of particular interest because they help clinicians make improved therapeutic decisions, including individualized risk prediction.^8^ An optimized decision-making process is particularly relevant for ECT patients, an especially vulnerable patient population undergoing a burdensome treatment. Fortunately, a plethora of studies addressing prognostic factors have been published in the past decade. However, they are often known to be poorly designed and reported.^8^ Therefore, meta-analyses can help to discern the strengths and weaknesses of these articles, resulting in a clear summary of evidence that aids the interpretation of the prognostic factor.

Other than recent publications by Tsujii et al^9^ and Feenstra et al,^10^ as far as is known, no articles have been published that systematically investigate prognostic factors for PIA. Furthermore, gaps remain even with these recent articles. Tsujii et al do not perform a meta-analysis and use the term “delirium” but define it broadly, including longer-lasting delirium and delirium that is assessed only after the whole ECT course in their definition. Feenstra et al do perform a meta-analysis, but the focus is limited to pharmacological prevention of PIA only. Therefore, a meta-analysis and systematic review that provides a proper evaluation of prognostic factors for PIA, namely, anesthesia-, patient-, and ECT-related, is necessary.

This systematic review and meta-analysis aims to accomplish 2 goals by identifying all studies that clearly define PIA and that report on prognostic factors for PIA. Specifically, the first goal is to identify anesthesia-, patient-, and ECT-related prognostic factors for PIA. The second goal is to provide a more exact PIA incidence by adhering to a strict definition of PIA and by using demographic and clinical characteristics. For both goals, the ultimate objective is to facilitate more informed clinical decision-making and to identify potential areas for future research.

## MATERIALS AND METHODS

This systematic review and meta-analysis follows the PRISMA (Preferred Reporting Items for Systematic reviews and Meta-analyses) checklist (http://links.lww.com/JECT/A225) for reporting.^11^ No protocol or registration was done, but the guide for systematic reviews and meta-analyses for prognostic factor studies was consulted for design and execution.^12^

A systematic search was conducted for the databases MEDLINE, EMBASE, Web of Science, and Cochrane, from inception until May 22, 2023, and was created by a practiced information scientist (see Supplemental Digital Content, Appendix S1, http://links.lww.com/JECT/A226).^13^ The search was limited to publications in English. Studies were eligible for inclusion when they reported the number or percentage of patients experiencing PIA objectively; ECT patients were 18 years or older; and prognostic factors for PIA could also be extracted. Randomized controlled trials were included when a power calculation was reported, and retrospective observational studies were included when the number of patients or ECT sessions to be analyzed was at least 150 (the smallest number reported in the included RCTs). Other study designs were excluded, namely, case reports, case series, literature reviews, conference papers, and trial registrations. Studies were also excluded when no full text was available, if they were published before 1980, or if they reported data that were collected in its entirety before 1980. Since the *Diagnostic and Statistical Manual of Mental Disorders, Third Edition* was released in this year, introducing a novel diagnostic standard in psychiatry, the 1980 cutoff was deemed necessary.^14^

Two independent reviewers (M.E., E.S.V.B.) screened all articles for eligibility for both the title/abstract and full-text phases. Disagreements between reviewers were resolved through a consensus meeting. If a consensus still could not be reached, a third reviewer (M.K.) was consulted for a final decision. The same 2 reviewers independently completed a risk-of-bias assessment for each included study using the Quality in Prognosis Studies (QUIPS) tool, and disagreements were resolved in the same manner.^15^

A study characteristics table and prognostic factor table were created to store extracted data from the included studies. The study characteristic table includes the following: first author, year, country, study design, inclusion period, number of analyzed participants, PIA definition, PIA cases, PIA cases based on patient/session, age, percent male sex, extracted prognostic factors, inclusion and exclusion criteria, anesthetic agent(s), and ECT electrode placement type. The prognostic factor table includes the following: first author, year, details related to the prognostic factor and PIA, PIA definition, number of PIA cases and noncases, exposed and unexposed to the prognostic factor, and the unadjusted effect measure with uncertainty.

When at least 5 studies reported the same PIA prognostic factor for which effect measures could be calculated, a meta-analysis was done. Due to the high heterogeneity in methods and outcomes between the included studies, a random-effects model was chosen in lieu of a fixed-effects model. The DerSimonian and Laird estimator was selected for the method, and both the 95% prediction interval and 95% confidence interval were included.^16^ The *Q* and *I*^2^ statistics were calculated to indicate the degree of heterogeneity. The pooled effect was also illustrated separately for observational studies and RCTs when both study types were present in one prognostic factor meta-analysis. Due to the limited number of studies in each prognostic factor meta-analysis, formal subgroup and sensitivity analyses were not carried out.^17^ All calculations and analyses were performed with R (version 4.3.0) using the Metafor package for R.^18^

When at least 2 studies reported the same PIA prognostic factor for which effect measures could be calculated, that factor was described in the prognostic factor tables without meta-analysis. In order to report effect measures for categorical and continuous prognostic factors, odds ratios and mean differences with standard deviation (SD) were calculated. When only the median and interquartile range were provided, they were subsequently converted into a mean and SD. When only the median (interquartile range) was reported, the mean (SD) was calculated from this.^19^ For each effect measure, the 95% confidence interval was calculated, and the direction of effect was illustrated.^20^

Finally, an informal descriptive comparison was conducted among studies that reported PIA incidence calculated based on the number of ECT sessions in which PIA occurred (session level). This comparison focused on assessing the resulting incidence when all studies were included, as opposed to when studies classified as having a high risk of bias in outcome measurement were excluded (adjusted incidence). The same comparisons were made for studies that reported PIA incidence calculated based on the number of patients experiencing it (patient level).

## RESULTS

**FIGURE 1:**
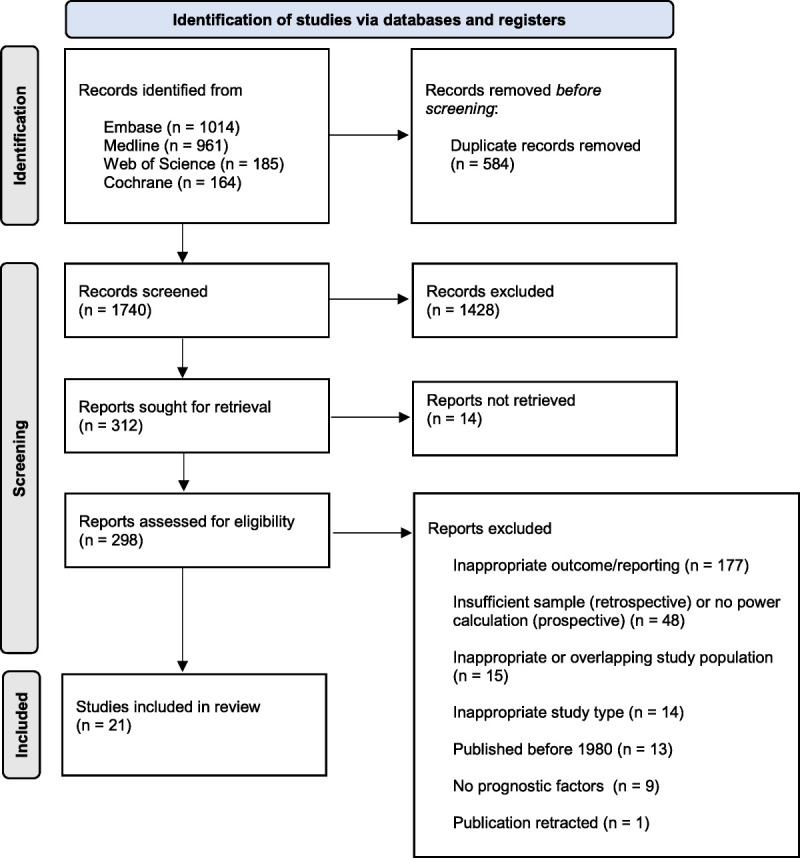
PRISMA flow diagram.

A total of 2324 studies were identified through a systematic search of the literature. After 584 duplicates were removed, 1740 articles remained. Of these, 1428 were excluded based on title and abstract, leaving 312 studies. Fourteen articles were not retrieved. That left 298 articles to be assessed with full-text screening. Finally, 21 studies remained that met the eligibility criteria and are included in the present article (Fig. 1).^21–41^ Of the 21 included studies, 10 (48%) were RCTs (5 of which were also crossover),^22,23,25,26,34,36,38–41^ and 11 (52%) were retrospective observational studies.^21,24,27–33,35,37^ There were 66,047 patients analyzed in total. Thirty-five percent were male, and the average age was 47.3 years. The complete list of study characteristics can be found in Supplemental Digital Content, Table S1 (http://links.lww.com/JECT/A227). A summary of a few characteristics is displayed in Table 1.

**TABLE 1:** Characteristics of Included Studies

	n (%)
**Geographic area**	
Asia	8 (38)
Asia-Europe	3 (14)
Europe	7 (33)
USA	3 (14)
**Study type**	
RCT	5 (24)
RCT (crossover)	5 (24)
Retrospective observational	11 (52)
**PIA reporting based on**	
Patient	10 (48)
Session	5 (24)
Both	6 (29)

**FIGURE 2:**
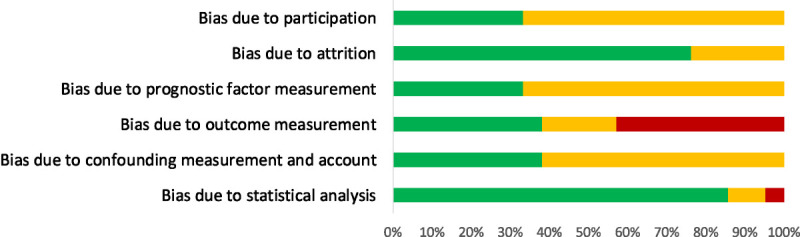
Results of the quality assessment. Risk of bias key: green/gray: low; yellow/light gray: moderate; red/dark gray: high.

The full QUIPS quality assessment can be found in Supplemental Digital Content, Table S2 (http://links.lww.com/JECT/A228). Figure 2 illustrates a summary of the quality assessment. A high risk of bias was found for 9 studies (43%) under the “outcome measurement” category and for one study (5%) under the “statistical analysis” category. The remaining studies had a low or moderate risk of bias for the remaining categories.

**FIGURE 3:**
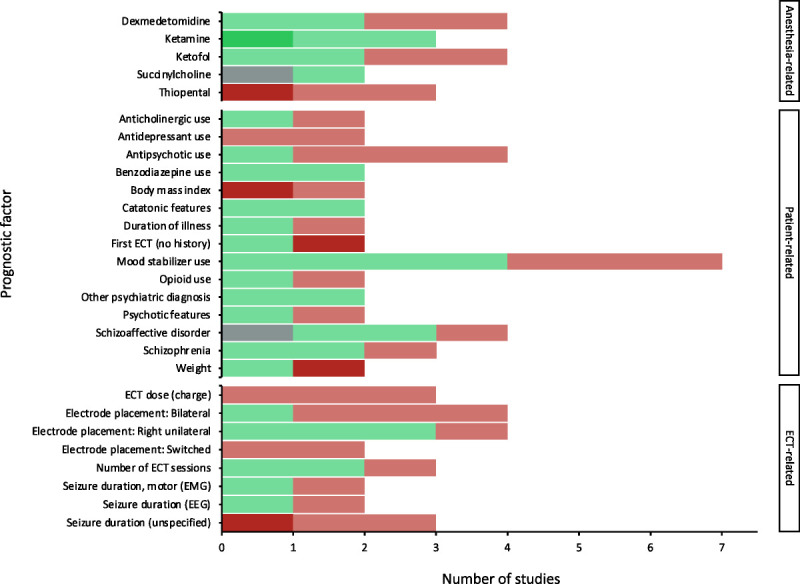
Direction of effect for prognostic factors not summarized with a meta-analysis. Dark green/gray: significant lower risk of PIA; light green/light gray: nonsignificant lower risk of PIA; dark red/black: significant higher risk of PIA; light red/dark gray: nonsignificant risk of PIA; gray/lightest gray: zero effect.

A total of 35 prognostic factors were identified for PIA after ECT, which are categorized into 8 anesthesia-related prognostic factors, 19 patient-related prognostic factors, and 8 ECT-related prognostic factors. A meta-analysis was conducted for 3 anesthesia-related, 4 patient-related, and zero ECT-related prognostic factors. The remaining prognostic factors were assessed for direction of effect only and can be found summarized in Figure 3. The details of each prognostic factor summarized without meta-analysis can be found in Supplemental Digital Content, Tables S3–S5 (http://links.lww.com/JECT/A229).

**FIGURE 4:**
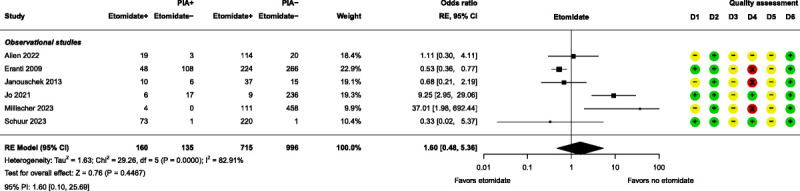
Forest plot for etomidate. D1, bias due to participation; D2, bias due to attrition; D3, bias due to prognostic factor measurement; D4, bias due to outcome measurement; D5, bias due to confounding measurement and account; D6, bias in statistical analysis and reporting. RE, random effects; PI, prediction interval.

**FIGURE 5:**
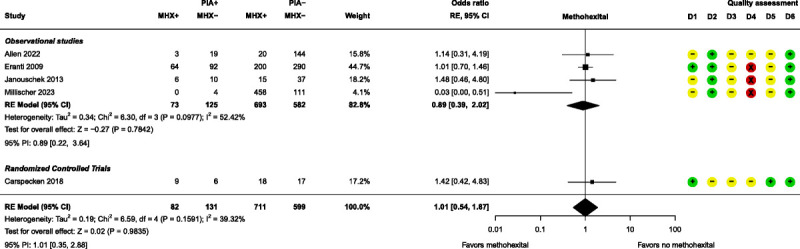
Forest plot for methohexital. D1–D6 as per Figure 4. MHX indicates methohexital.

**FIGURE 6:**
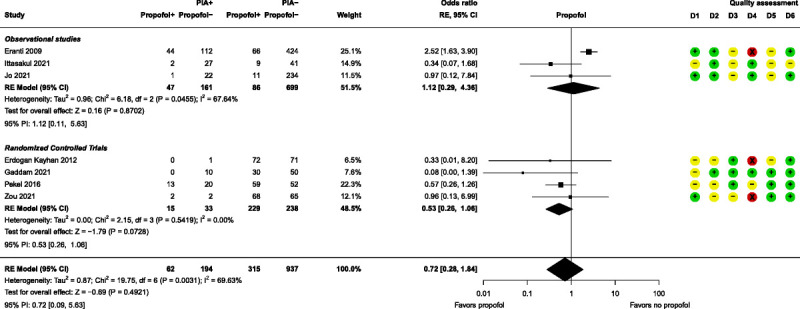
Forest plot for propofol. D1–D6 as per Figure 4.

Meta-analyses were performed for 3 anesthesia-related prognostic factors, namely, etomidate, methohexital, and propofol. None of these factors had a significant effect on PIA (Figs. 4–6). Further details of these 3 factors can be found in Supplemental Digital Content, Table S6 (http://links.lww.com/JECT/A230). Five anesthesia-related prognostic factors were summarized with the directions of effect only. Only ketamine was shown to be in the direction of decreasing the risk of PIA and thiopental in the direction of increasing the risk of PIA. The rest of the anesthesia-related prognostic factors showed directions of effect that were inconsistent.

**FIGURE 7:**
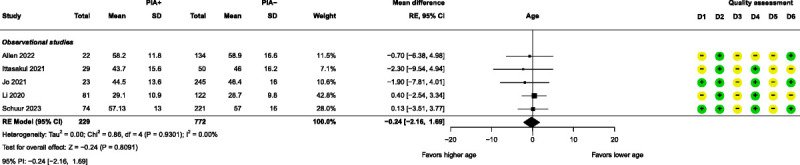
Forest plot for age. D1–D6 as per Figure 4.

**FIGURE 8:**
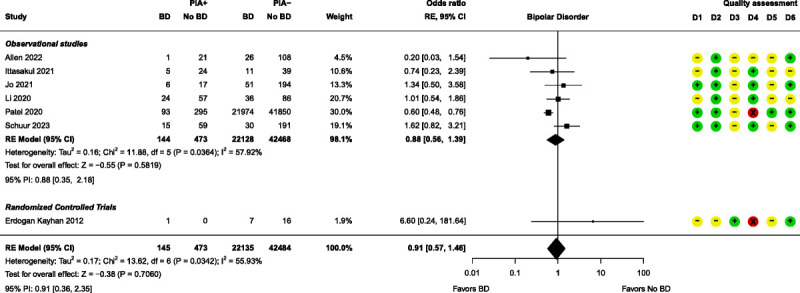
Forest plot for bipolar disorder. D1–D6 as per Figure 4. BD indicates bipolar disorder.

**FIGURE 9:**
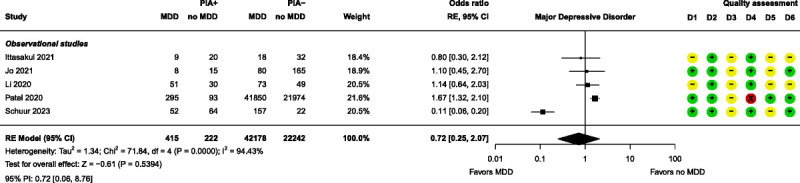
Forest plot for MDD. D1–D6 as per Figure 4.

**FIGURE 10:**
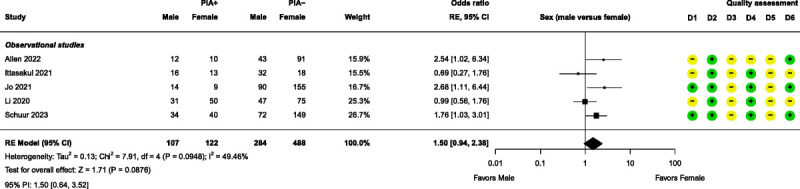
Forest plot for sex. D1–D6 as per Figure 4.

Meta-analyses were performed for 4 patient-related prognostic factors, namely, age, bipolar disorder, major depressive disorder (MDD), and sex. None of these factors had a significant effect on PIA (Figs. 7–10). Further details of these 4 factors can be found in Supplemental Digital Content, Table S7 (http://links.lww.com/JECT/A231). Fifteen patient-related prognostic factors were summarized with direction of effect only. The studies reporting antidepressant use, body mass index (BMI), and ECT history were all shown to be in the direction of increasing the risk of PIA. The studies reporting benzodiazepine use, catatonic features, and a psychiatric diagnosis of “other” (ie, other than MDD, bipolar disorder, schizophrenia, or schizoaffective disorder) were all shown to be in the direction of decreasing the risk of PIA. The rest of the patient-related prognostic factors showed directions of effect that were inconsistent.

Eight ECT-related prognostic factors were summarized with direction of effect. The studies reporting ECT dose (charge), electrode placement (switched), and seizure duration (unspecified) were all shown to be in the direction of increasing the risk of PIA. The rest of the ECT-related prognostic factors showed directions of effect that were inconsistent.

Incidence of PIA was determined for studies reporting PIA at the patient level and can be seen in Table 2. Of 21 studies, 16 reported PIA at the patient level. The average PIA incidence for these studies is 13.9%. Of these 16, 7 have a high risk of bias in the “outcome measurement” category of the risk-of-bias assessment. When removing these studies and recalculating PIA incidence, the resulting incidence is 18.0%.

**TABLE 2:** Incidence for PIA Measured at the Patient Level

Authors	Year	Country	PIA+	PIA−	PIA %	D4 High Risk
Allen et al^20^	2022	USA	22	134	14.1%	No
Bagle et al^21^	2016	India	3	57	5.0%	No
Carspecken et al^22^	2018	USA	15	35	30.0%	No
Erdogan Kayhan et al^24^	2012	Turkey	1	23	4.2%	Yes
Gazdag et al^26^	2006	Hungary	5	37	11.9%	Yes
Ittasakul et al^27^	2021	Thailand	29	50	36.7%	No
Janouschek et al^28^	2013	Germany	16	52	23.5%	Yes
Jo et al^29^	2021	Korea	23	245	8.6%	No
Kranaster et al^30^	2011	Germany	4	38	9.5%	Yes
Li et al^31^	2020	China	81	122	39.9%	No
Parikh et al^33^	2017	India	0	30	0.0%	No
Patel et al^34^	2020	USA	388	63,824	0.6%	Yes
Schuur et al^36^	2023	The Netherlands	74	221	25.1%	No
Semkovska et al^37^	2016	Ireland	3	135	2.2%	No
Yeter et al^39^	2022	Turkey	5	55	8.3%	Yes
Zou et al^40^	2021	China	4	133	2.9%	Yes
Mean PIA incidence					13.9%	
Mean PIA when removing D4 high risk = yes					18.0%	

D4, bias due to outcome measurement.

Incidence of PIA was also determined for studies reporting PIA at the session level and can be seen in Table 3. Of 21 studies, 11 reported PIA at the session level. The average PIA incidence for these studies is 12.4%. Of these 11, 5 have a high risk of bias in the “outcome measurement” category of the risk-of-bias assessment. When removing these studies and recalculating PIA incidence, the resulting incidence is 16.5%.

**TABLE 3:** Incidence for PIA Measured at the Session Level

Authors	Year	Country	PIA+	PIA−	PIA %	D4 High Risk
Carspecken et al^23^	2018	USA	41	256	13.8%	No
Eranti et al^24^	2009	UK	156	490	24.1%	Yes
Erdogan Kayhan et al^25^	2012	Turkey	1	143	0.7%	Yes
Gaddam et al^26^	2021	India	10	80	11.1%	No
Ittasakul et al^28^	2021	Thailand	86	826	9.4%	No
Janouschek et al^29^	2013	Germany	24	712	3.3%	Yes
Jo et al^30^	2021	Korea	23	245	8.6%	No
Millischer et al^33^	2023	Austria	4	569	0.7%	Yes
Pekel et al^36^	2016	Turkey	33	111	22.9%	No
Subsoontorn et al^39^	2021	Thailand	24	48	33.3%	No
Yeter et al^40^	2022	Turkey	5	55	8.3%	Yes
Mean PIA incidence					12.4%	
Mean PIA when removing D4 high risk = yes					16.5%	

D4, bias due to outcome measurement.

## DISCUSSION

This systematic review and meta-analysis was carried out to accomplish 2 main goals: to identify and summarize the evidence for anesthesia-, patient-, and ECT-related prognostic factors for PIA and to elucidate the diverse incidences of PIA based on demographic and clinical characteristics.

Our results regarding the first objective indicate that none of the prognostic factors demonstrated a significant effect on reducing or increasing PIA incidence, although several prognostic factors showed a consistent direction of effect and would be worth investigating further in future studies. Furthermore, the majority of the risk-of-bias domains were categorized as having low or moderate rates of risk. However, around 40% of the studies had a high risk of bias in the outcome measurement domain.

### Anesthesia-Related Prognostic Factors

All 3 anesthesia-related prognostic factor meta-analyses, namely, etomidate, methohexital, and propofol, resulted in nonsignificant pooled results as prognostic factors for PIA. For each anesthetic agent, the result is moderately to highly heterogeneous, making it difficult to draw definitive conclusions. This is unsurprising due to differences in PIA severity, number of ECT sessions, age, and comparator anesthetics. For etomidate as prognostic factor, 3 studies only recorded PIA when it was severe,^21,30,37^ as did 2 studies for methohexital^21,23^ and propofol^28,30^ in their respective analyses. Jo et al^30^ included only the first ECT session in their analysis, whereas the other studies included multiple sessions and investigated maintenance ECT, which may not necessarily equate with regular ECT courses.^33^ Age differences can be seen between Zou et al, who included only elderly patients,^41^ and participants in Erdogan Kayhan et al, where the average age was 26.^25^ Even within studies this can occur, such as Eranti et al, where the propofol group had significantly older participants compared with those in the other anesthetic groups.^24^ Finally, variety in comparator anesthetics is also widespread. Etomidate was compared in different studies to methohexital,^21,24,29,33^ propofol,^24,30^ pentobarbital,^30^ and unknown other anesthetic.^37^ Methohexital was compared with etomidate,^21,24,29,33^ propofol,^24^ and ketamine,^23^ and propofol was compared with etomidate,^24,30^ methohexital,^24^ ketofol,^25,26,41^ thiopental,^26,28^ pentobarbital,^30^ and sevoflurane.^36^

Of particular interest is the nonsignificant result concerning propofol, given its frequent mention as a treatment or preventively measure for PIA that appears regularly in the literature,^5,42,43^ including in a systematic review that cites Gaddam et al^26^ and Tzabazis et al.^6^ In these articles, propofol use led to reduced PIA occurrences, in contrast to the nonsignificant pooled result of this meta-analysis. Considering the conflicting evidence, further exploration of propofol as a PIA prognostic factor is advised.

It is clear that more high-quality studies should be carried out to investigate etomidate, methohexital, and propofol use as prognostic factors for PIA, as 3 studies in each meta-analysis were categorized as high risk of bias in outcome measurement (measurement of PIA). Unfortunately, it is not recommended to do a sensitivity analysis with fewer than 10 studies,^17^ but for future investigation, an additional analysis where this type of study would be removed is advised.

### Patient-Related Prognostic Factors

All 4 patient-related prognostic factor meta-analyses, namely, age, bipolar disorder, MDD, and sex, resulted in nonsignificant pooled results as prognostic factors for PIA. Except for age, the meta-analyses also demonstrated moderate to high heterogeneity. With respect to age, the nonsignificant results contradicted a publication by Andrade et al, where old age is said to be a risk factor for the development of PIA.^1^ This might be explained by the fact that the average ages in all the combined studies range from about 29 to 59 years, which falls short of representing a genuinely elderly population (>65 years old) to establish a significant effect of age on PIA incidence. However, the nonsignificant result is in line with the findings of other studies.^2,5,44^

Except for the individual study results from Patel et al, the nonsignificant bipolar disorder and MDD meta-analysis results were in line with the results of the studies from which they are made, as well as others.^2,21,25,28,30,32,35,37^ Bipolar disorder and MDD often come up as potential prognostic factors for PIA but fairly consistently show no effect. This could be due to lithium utilization and/or presence of catatonic features, frequently observed in conjunction with MDD and bipolar disorder. Because both lithium use and catatonic features have been recognized as prognostic factors that contribute to a higher incidence of PIA, their additional presence may have led to the erroneous identification of these disorders as prognostic indicators.^5,45,46^ However, this is merely conjecture and should be explored further. Moreover, despite the existence of various subtypes within both disorders, the literature often simplifies them to “bipolar disorder” and “MDD” or “depression.” Consequently, a possible effect may stay hidden, necessitating more nuanced research to elucidate the roles of bipolar disorder and MDD on PIA outcomes.

Finally, the nonsignificant sex meta-analysis contradicted the anticipated trend, with 3 of 5 studies suggesting males experienced significantly more PIA in univariate analyses.^21,30,37^ However, heterogeneity is only moderate, and none of the studies have a high risk of bias in any of the 6 domains, so locating the reason for the divergence from the expected result proves more difficult. Notably, though, male sex lost significance after multiple testing correction or multivariate analysis in the 3 studies, consistent with recent publications.^2,5^

### Prognostic Factors Not Summarized With Meta-analysis

Of the 5 anesthesia-related prognostic factors summarized with directions of effect, only ketamine was shown to be in the direction of decreasing and thiopental in the direction of increasing the risk of PIA. In the studies evaluating ketamine, the comparator anesthetics were thiopental and methohexital. Comparing ketamine with these induction agents, rather than with propofol, might elucidate its potential in reducing risk, given propofol's historical use in managing agitation.^47^ Therefore, it would be worthwhile to conduct future studies comparing the risk of PIA between ketamine and propofol as induction agents. Thiopental may be of similar interest in that it has not been assessed in the literature often,^10,26^ yet 3 separate studies in the present review found that it increased the risk of PIA, one even significantly.

Of the 15 patient-related prognostic factors, antidepressant use and BMI were shown to be in the direction of increasing risk of PIA, whereas benzodiazepine use, catatonic features, and a psychiatric diagnosis of “other” were relevant for decreasing risk of PIA. Body mass index has seldom been explored for its relation to PIA, being left out of a recent systematic review entirely.^9^ However, Ittasakul et al found a significantly increased PIA risk for patients with a high BMI. They suggest this may be due to the high occurrence of oxygen desaturation in this population, but this needs further investigation.^28^ Because benzodiazepines are typically administered to mitigate PIA in clinical practice, the direction of decreasing risk was anticipated. In contrast, the direction of decreasing risk for catatonic features was a surprising result, considering catatonia has previously been found to increase the risk of PIA.^5^

Of the 8 ECT-related prognostic factors, ECT dose (charge), electrode placement (switched), and seizure duration (unspecified) were in the direction of increasing risk. ECT parameters used as prognostic factors for PIA is a highly debated topic. As the direction of effect also demonstrated, additional studies suggest that the higher ECT dose and a longer seizure duration increase the risk for PIA.^1,2^ However, others have found no significant indication that this is the case.^28,44^ It is a similar story with electrode placement.^1,3,9,28^ However, it is not uncommon in ECT treatment to start with unilateral and switch to bilateral when the seizure or clinical response is insufficient at the highest possible unilateral ECT dose.^23^ Because higher ECT dose may be related to PIA, this might explain the increased direction of effect for switched electrode placement. However, this is not a significant finding, and regardless, further evaluation of these factors in future studies is advised.

The second goal was to estimate and elucidate PIA incidence, and our results emphasize the importance of calculating and interpreting incidence at the patient and session levels separately. Moreover, considering risk of bias is crucial for obtaining accurate incidence rates, just as it is for evaluating prognostic factors.

### Estimating Postictal Agitation Incidence

In addition to considering the number of patients who experience PIA, the number of sessions is also important to take into consideration when analyzing data reporting PIA incidence, as 34% of patients who have experienced PIA may experience it again.^48^ Therefore, overall incidence may decrease after several sessions compared with the first session. Furthermore, some clinicians may preventively administer a sedative for future ECT sessions, potentially leading to an underestimation of the true incidence (as described in the methods of Carspecken et al).^23^ This creates a challenge in approximating a representative PIA incidence unless demographic and clinical characteristics, as well as protocols, are scrutinized. This pattern can also be seen in the descriptive results of PIA incidence. The average incidence reported at the patient level is 13.9% compared with 12.4% reported at the session level. Although this is a seemingly small separation, differences between patient- and session-level incidence rates can determine whether a prognostic factor shows significance or not. For example, Carspecken et al report PIA based on session and based on patient separately, and only the latter shows nonsignificance and is included in the present meta-analysis for methohexital. This mismatch in significance nicely illustrates why separating PIA incidence based on patient and session is important for interpreting results.

Other factors may also impact PIA incidence, such as the method of assessment, or lack of. PIA can be measured with a scale, such as the Richmond Agitation Sedation Scale^49^; it can be defined according to high levels of agitation that require sedative administration, or it can be diagnosed by a clinician based on their professional judgment. This last category may lead to false-negative results, especially when noting PIA in the patient's chart is not common clinical practice. Therefore, PIA reporting based on a scale or pharmacological intervention was considered more accurate and had a lower risk of bias, comparatively. Our results also indicated possible false-positives: the mean PIA incidences jump to 18.0% and 16.5%, at the patient versus session level, respectively, after removing studies with a high risk of bias in the outcome assessment domain.

### Strengths, Limitations, and Future Direction

This study has several strengths. First, this is the first meta-analysis to report on prognostic factors for PIA after ECT that at the same time assesses PIA incidence at the patient and session levels. Second, the highly specific definition of PIA encompassing both specific features and timing criteria facilitated an easier comparison of results across the included studies, making drawing conclusions more straightforward. This contrasts with a similar systematic review where a looser definition was mentioned as a limitation.^9^ Similarly, including only studies published after 1980, and if a prospective RCT, reporting a power calculation, led to a higher-quality final group of included articles. Finally, a narrowed, nuanced, and more reliable PIA incidence was clarified at both the patient and session levels. These results can serve as valuable references for researchers and clinicians alike. It is recommended to utilize the findings obtained after omitting articles with a high risk of bias in outcome measurement; however, it is crucial to consider factors specific to the particular patient population under investigation as well.

This study also has some limitations that should be noted. Although strict inclusion criteria yield a selection of higher-quality and more comparable articles, the limited number of eligible studies may restrict the ability to engage in meaningful discussion and analysis. This limitation was evident in the meta-analyses where heterogeneity was often high: conducting subgroup and sensitivity analyses to identify potential sources of this heterogeneity was not feasible. Furthermore, although strict inclusion criteria supposedly increased the overall quality of included studies, 43% of the articles had a high risk of bias in outcome measurement. Finally, it was not possible to perform meta-analyses for several potential prognostic factors due to a limited number of studies reporting on them, so this could be a direction for future research.

Considering the nonsignificant findings for 7 PIA prognostic factors in this meta-analysis, several areas warrant further investigation. First, future research should focus more on exploring potential sources of heterogeneity through conducting subgroup and sensitivity analyses with several factors in mind, such as variations in study design, patient characteristics (eg, diagnoses), and assessment methods (ie, tool vs clinical judgment). Additionally, efforts should be made to improve the quality of outcome measurement in studies examining PIA. Reducing bias through the utilization of standardized and validated assessment tools and implementing rigorous methodology, ideally with RCTs, would enhance the reliability and validity of future findings. Furthermore, although the current analysis does not delve into aspects such as magnetic resonance imaging and brain abnormalities, they have been of interest in previous studies.^9,37^ Other potential considerations for future research include, but are not limited to, intracranial pressure, electrolyte imbalance, and endocrine disorders. Similarly, conducting additional meta-analyses to explore potential prognostic factors identified in the present analysis, such as investigating the impact of ketamine, thiopental, and various ECT parameters (ie, dose, electrode placement, seizure duration), could provide valuable insights into clarifying their roles as potential prognostic factors. Finally, we emphasize the importance of an open and collaborative discussion to achieve a consensus on a standardized definition and measurement procedure for PIA, a vital step for guiding future research. This concerted effort will undoubtedly improve the accuracy of assessments and interventions related to PIA, ultimately benefiting the affected patients.

## CONCLUSION

The current review has presented several proposed prognostic factors for PIA after ECT. Although none of the prognostic factors investigated with a meta-analysis were found to be significant, several prognostic factors, namely, ketamine, thiopental, antidepressant, and benzodiazepine use; catatonic features; other psychiatric diagnosis; ECT dose (charge); electrode placement: switched; and seizure duration (unspecified), showed a consistent direction of effect and provide direction for future research. This is especially valuable due to limited high-quality data on this issue, which consequently complicates the task of drawing definitive conclusions and making recommendations to clinicians to improve ECT. Finally, our identification of adjusted mean PIA incidences of 18.0% and 16.5% at the patient and session levels, respectively, serves as a valuable reference for researchers and clinicians alike. We hope this review can initiate a discussion within the scientific community, leading to a consensus on adopting a standardized definition and rigorous diagnostic procedure for PIA.
